# A model for determining cardiac mitochondrial substrate utilisation using stable ^13^C-labelled metabolites

**DOI:** 10.1007/s11306-019-1618-y

**Published:** 2019-11-26

**Authors:** Ross T. Lindsay, Demetris Demetriou, Dominic Manetta-Jones, James A. West, Andrew J. Murray, Julian L. Griffin

**Affiliations:** 1 Department of Biochemistry and the Cambridge Systems Biology Centre, University of Cambridge, Cambridge, UK; 2 Department of Physiology, Development and Neuroscience, University of Cambridge, Cambridge, UK; 3 Department of Engineering, University of Cambridge, Cambridge, UK

**Keywords:** Krebs cycle, TCA cycle, Isotopomer analysis, Fluxomics, Metabolic substrate switching, LC–MS/MS, Heart

## Abstract

**Introduction:**

Relative oxidation of different metabolic substrates in the heart varies both physiologically and pathologically, in order to meet metabolic demands under different circumstances. ^13^C labelled substrates have become a key tool for studying substrate use—yet an accurate model is required to analyse the complex data produced as these substrates become incorporated into the Krebs cycle.

**Objectives:**

We aimed to generate a network model for the quantitative analysis of Krebs cycle intermediate isotopologue distributions measured by mass spectrometry, to determine the ^13^C labelled proportion of acetyl-CoA entering the Krebs cycle.

**Methods:**

A model was generated, and validated ex vivo using isotopic distributions measured from isolated hearts perfused with buffer containing 11 mM glucose in total, with varying fractions of universally labelled with ^13^C. The model was then employed to determine the relative oxidation of glucose and triacylglycerol by hearts perfused with 11 mM glucose and 0.4 mM equivalent Intralipid (a triacylglycerol mixture).

**Results:**

The contribution of glucose to Krebs cycle oxidation was measured to be 79.1 ± 0.9%, independent of the fraction of buffer glucose which was U-^13^C labelled, or of which Krebs cycle intermediate was assessed. In the presence of Intralipid, glucose and triglyceride were determined to contribute 58 ± 3.6% and 35.6 ± 0.8% of acetyl-CoA entering the Krebs cycle, respectively.

**Conclusion:**

These results demonstrate the accuracy of a functional model of Krebs cycle metabolism, which can allow quantitative determination of the effects of therapeutics and pathology on cardiac substrate metabolism.

**Electronic supplementary material:**

The online version of this article (10.1007/s11306-019-1618-y) contains supplementary material, which is available to authorized users.

## Introduction

Stable isotope labelling has been an invaluable tool for the study of metabolic fluxes. An important use of ^13^C labelled metabolites has been to investigate the proportional contribution of different substrates to the Krebs cycle under varying conditions and across different tissues. This has generated many insights, particularly when used in studies of the isolated perfused heart where there can be significant changes in substrate selection under different pathological and physiological conditions (Aubert et al. 2016; Crown et al. 2016; Liu et al. 2016; Lloyd et al. 2003, 2004). Interpretation of the isotopic distribution of Krebs cycle intermediates arising in such studies is far from trivial, however, and a reliable model is therefore required.

Mass spectrometry is a very sensitive approach for measuring the concentrations of Krebs cycle intermediates (Katzs et al. 1993; Taegtmeyer et al. 2016; Yang et al. 2008). The proportion of each intermediate which is 1–6 units heavier as a result of ^13^C labelling is also directly detectable, and thus, differences in the proportion of ^13^C labelled metabolic substrate oxidation are detected in the labelling patterns. The complexity of carbon flux in the Krebs cycle means a model is required to accurately assign a given labelling pattern to a proportion of labelled substrate oxidation, particularly in situations where multiple substrates may be present. There have been multiple offerings for large scale models of cardiac metabolism, as reviewed in Taegtmeyer et al. (2016). Briefly, whilst large scale stoichiometric models consider the proportional metabolism of compounds into their products, kinetic models employ information about the rate constants of the enzymes involved to determine flux. However, for a simple and easily applicable analysis of isotopologue distribution data, it is important to consider not only the flux of molecules through enzymes, but also migration of labelled carbons within the molecules themselves. Therefore, we propose an easy to apply network model which accounts for both the stoichiometry between molecules in the network, and also that between isotopomers.

To generate such a model, there are several considerations which must be taken into account. ^13^C originating from metabolism of U^13^C labelled fatty acids and carbohydrates primarily enters the Krebs cycle as [1,2-^13^C_2_]acetyl-CoA. However, stereochemistry and the cyclic nature of the pathway leads to a complex distribution of isotopomers (molecules with equivalent numbers of labelled and unlabelled carbon atoms, but differing in their positions) and isotopologues (molecules with different numbers of labelled and unlabelled carbons) at steady state. Incorporation of 2 + amu acetyl-CoA (labelled with two ^13^C atoms) into the Krebs cycle to initially form a 2 + amu citrate isotopologue results in the generation of a greater number of isotopologues of succinate, malate and other intermediates through subsequent cycles than can be accounted for by considering 2 + amu intermediates alone. Indeed, the full range of citrate isotopologues with 1–6 + amu can all result from an input of [1,2-^13^C] acetyl-CoA.

The cyclic nature of the Krebs cycle (Krebs et al. 1938) thus leads to amplification of labelling at equilibrium. Whilst the greatest mass increase an intermediate could gain from a single turn of the cycle with the input of [1,2-^13^C_2_] acetyl-CoA would be 2 + amu, the resulting 2 + amu oxaloacetate can yield 2 + and 4 + labelled citrate when combined with unlabelled and [1,2-^13^C_2_]acetyl-CoA, respectively. Over a number of cycles towards steady state, this results in an array of isotopologues.

**Fig. 1:**
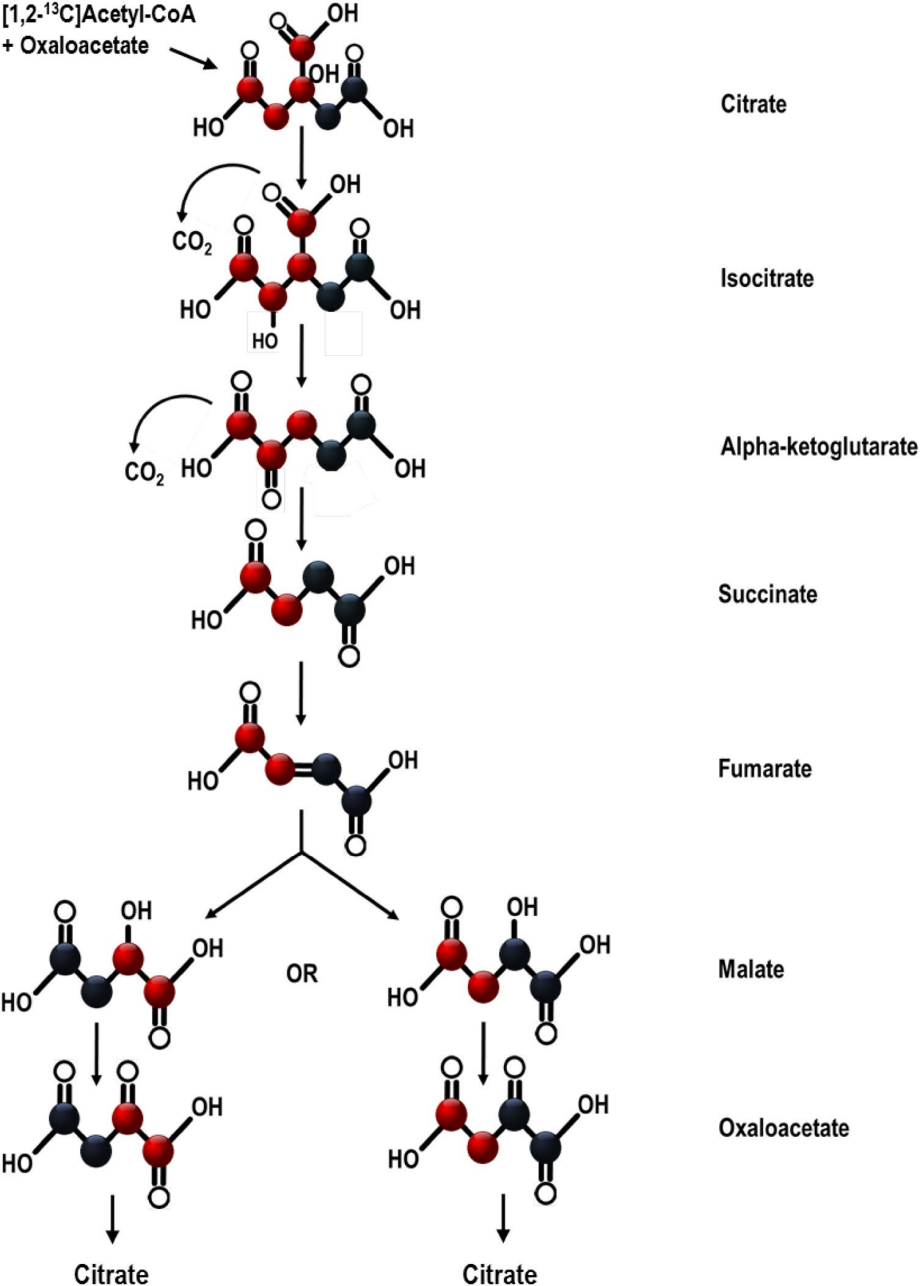
Movement of labelled carbons during one turn of the Krebs cycle. Black represents a ^13^C labelled carbon, and red represents an unlabelled one. Labelled citrate with + 2 amu, formed from oxaloacetate and [1,2-^13^C]acetyl-CoA, is equally likely to produce one of two different isotopologues of oxaloacetate with + 2 amu

Furthermore, two carbons are removed as CO_2_ during each turn of the cycle based upon their position in isocitrate and α-ketoglutarate, and indiscriminately of atomic mass (Krebs et al. 1938). Since fumarate is non-enantiomeric, [1,2-^13^C]fumarate is equally likely to become 1,2 or 3,4 ^13^C labelled malate when hydrated across its double bond (Fig. 1). When acetyl-CoA reacts with the resulting oxaloacetate, one ^13^C carbon from [1,2-^13^C]oxaloacetate will arrive in a position on isocitrate where it is to be lost as ^13^CO_2_, whilst one ^13^C from [3,4-^13^C]oxaloacetate will be removed by α-ketoglutarate dehydrogenase. The outcome of this is that a single ^13^C is lost from some labelled intermediates, and the position of the remaining ^13^C varies, thereby affecting its future fate. This further complicates the distribution of isotopologues produced at steady state.

Here we present a mathematical model which takes the full complexity of the Krebs cycle into account. The model can map ^13^C accumulation within the cycle to steady state and has been used to calculate the relative oxidation of different metabolites in a perfused heart system.

## Methods

### Animal work

Rats used in this study were euthanised using an approved Schedule 1 method by a competent, personal licence holder in accordance with the Animals in Scientific Procedures Act 1986 and UK Home Office guidance. Animals were given standard chow and water ad libitum and housed in a 12 h/12 h light/dark cycle.

### Materials

All reagents and chemicals were obtained from Sigma-Aldrich Ltd (UK) unless otherwise stated.

### Generation of the model

#### Initial conditions

The initial proportion of ^13^C labelled Krebs cycle intermediates before the administration of any label was taken to be 0, and the proportion of entirely unlabelled intermediates 1. The proportion of the acetyl-CoA input which was [1,2-^13^C_2_] acetyl-CoA was designated α.

##### Mapping the relationships of Krebs cycle isotopomers

The initial iterations of the Krebs cycle were worked through until every isotopomer possible for each intermediate had been introduced, and all were assigned a tag (C1, C2,… for citrate; A1, A2,… for α-ketoglutarate etc.). Four metabolites were crucial to the model—the 6-carbon intermediate citrate; the 5-carbon intermediate α-ketoglutarate; the non-stereochemical 4-carbon intermediate succinate, and the stereochemical 4-carbon intermediate malate. The isotopologue or isotopomer distributions of each of the other intermediates would be identical to one of these other metabolites, and therefore were not incorporated into the model for the purpose of calculating enrichment.

The relationship between each isotopomer at time T to the isotopomers of the preceding ‘crucial’ metabolite in the cycle at time T − 1 were drawn up as a set of differential equations. For example, if 1 + malate labelled on the alpha-carbon is designated *M*_1_, *M*_1_(T) = 0.5 *S*_6_ (T − 1), where *S*_6_ corresponds to the 1  + succinate isotopomer labelled on the carbon at the end of the chain. The fate of the 50% of *S*_6_ not converted to *M*_1_ is to become 1 + malate labelled at the carbon on the opposite end of the chain to the α-carbon, which we can designate *M*_2_ to get the equation *M*_2_(T) = 0.5 *S*_6_ (T − 1) and so on. Atom transitions for the metabolic network adhering to the guidelines of Crown and Antoniewicz (2013) can be found in Supplementary Tables 1–3.

Next, a network model was generated. This was achieved by multiplying a 1 by 78 vector comprising the 78 isotopomers of citrate, α-ketoglutarate, succinate and malate at time T with a 78 by 78 network matrix comprising the relationships of those isotopomers to each other.

The model then assigned values for α to experimentally-obtained data by running iterations to determine which value of α corresponded to the smallest discrepancy between predicted and experimentally-measured isotopic distributions. This discrepancy score has been detailed for each α value presented in this paper, where a discrepancy score of 0 represents the perfect fit, and 1 the worst possible fit.

### Validation of the model in the isolated rat heart

#### Perfusion protocols

Male Wistar rats (300–350 g, ~ 9 weeks old) were obtained from a commercial breeder (Charles River, UK). Rats were euthanised by rising CO_2_ concentrations, followed by cervical dislocation.

Hearts were excised and perfused in the Langendorff mode at 38 °C and a constant pressure of 100 mmHg for 32 min, following an initial acclimatisation period. This time period is sufficient for carbon labelling in the perfused heart to reach steady state as shown by others previously (Chatham et al. 1995; Khairallah et al. 2004; Lewandowski and Hulbert 1991). We recognise that all isolated heart preparations are subtly different, and the possibility exists that ours may require more time to reach steady state compared to those in the aforementioned studies. However, given the similarity of our preparation to those mentioned, we have made the assumption that our preparation reached metabolic steady state in a similar time period. The perfusion medium was 250 mL of recirculating Krebs–Henseleit (KH) buffer with the basic composition 118 mM NaCl, 4.7 mM KCl, 1.2 mM MgSO_4_, 1.3 mM CaCl_2_, 0.5 mM EDTA, 25 mM NaHCO_3_ and 1.2 mM KH_2_PO_4_. The KH buffer was pH 7.4, and gassed with 95% O_2_, 5% CO_2_.

To validate that α determination was consistent when input percentages of U^13^C labelled glucose differed, the buffer was supplemented with 11 mM glucose. In separate experiments, 0%, 25%, 50%, 75% or 100% of this glucose was U^13^C labelled, while the remaining percentage remained unlabelled (n = 3 per condition).

Relative input of triacylglycerol and glucose from the perfusion buffer to the Krebs cycle was then determined in hearts perfused with both 11 mM glucose and 0.4 mM equivalent triacylglycerol mixture in the form of Intralipid, which would yield 1.2 mM fatty acid and 0.4 mM glycerol upon hydrolysis (composition of Intralipid detailed in Supplementary Table 4). The KH buffer contained either 8.25 mM unlabelled glucose, 2.75 mM U-^13^C labelled glucose (99% enrichment, Cambridge Isotopes Laboratories) and 0.4 mM Intralipid (n = 6); or 11 mM unlabelled glucose, 0.1 mM U-^13^C mixed triglycerides (99.6% enrichment Cambridge Isotopes Laboratories) and 0.3 mM unlabelled triacylglycerol in the form of Intralipid (n = 6).

At the end of the perfusion protocol, the left ventricle was rapidly sectioned in transverse and snap frozen for analysis by LC–MS/MS.

### Liquid chromatography-coupled mass spectrometry (LC–MS)

Isotopologue distributions of malate, citrate, glutamate (the cellular pool of which rapidly interconverts with α-ketoglutarate) and succinate were determined using LC–MS according to the MSI standards detailed in Supplementary Table 5.

Metabolites were extracted from heart samples using a methanol/chloroform/water extraction method as described previously (Le Belle et al. 2002). The resulting aqueous and organic phases were collected and dried by evacuated centrifuge before reconstitution in 10 mM ammonium acetate/internal standard mixture (200 µL; phenylalanine d5, Valine d8, leucine d10). Samples were run for 6 min on a Thermo Dionex Ultimate 3000 LC system using an ACE Excel-2 C18-PFP 5 μm column (100 A, 150 × 2.1 mm, 30 °C). Mobile phase A was 0.1% formic acid, while mobile phase B was acetonitrile plus 0.1% formic acid. The LC gradient was as follows: 0% B for 1.6 min followed by a linear gradient up to 30% B for 2.4 min. Following a further linear increase to 90% B over 30 s, B was held at 90% for 30 s before 1.5 min re-equilibration.

The LC system was coupled to a Thermo Elite orbitrap mass spectrometer, on which run parameters were as follows: Heater temp 420 °C, sheath gas flow rate 60 units, Aux gas flow rate 20 units, Sweep gas flow rate 5 units. The spray voltage was − 2.5, capillary temp 380 °C and S-lens RF level 60%. The raw peak areas obtained from mass spectra were inputted to the model without correcting for natural isotopologue abundance, as this natural abundance varies by an unknown factor which is in inverse proportion to the unknown value α.

Glutamate was measured as a proxy for α-ketoglutarate. Glutamate is interconverted rapidly with α-ketoglutarate and therefore their labelling distributions are identical (Chatham et al. 1995; Lewandowski et al. 1996; Melo et al. 2011; Yu et al. 1995).

The 6 + amu isotopologue of citrate also had a high limit of detection, and therefore in conditions where the 6 + amu isotopologue of citrate was expected citrate has not been considered in the fitting of the model.

### Statistical analysis

All results are displayed as mean ± SEM. 2-way ANOVA was employed with the percentage of buffer glucose which was U-^13^C labelled as one factor and the metabolite assessed as the other, to determine whether either factor affected the proportion of total oxidation which the model calculated to be accounted for by glucose oxidation.

## Results and discussion

### Prediction of isotopologue distribution in Krebs cycle metabolites

**Fig. 2:**
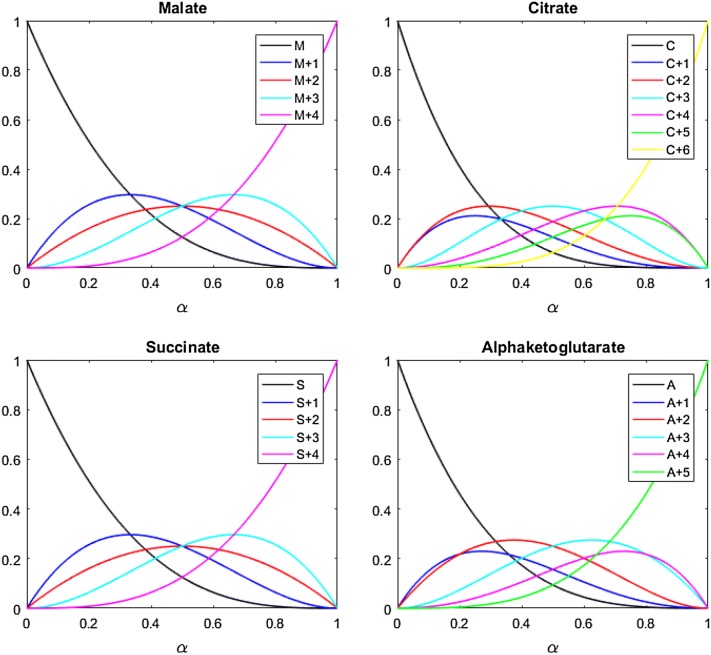
Bifurcation plots showing how the relative proportions of key Krebs cycle intermediate isotopologues vary at equilibrium as the proportion of [1,2-^13^C_2_]acetyl-CoA entering the Krebs cycle (α) increases, as predicted by the model

The model was used to predict isotopologue distributions of malate, citrate, α-ketoglutarate and succinate at every value of α between 0 and 1. The predicted proportion of each intermediate’s total cellular pool accounted for by each isotopologue at each value of α was visualised by plotting α against the predicted steady state distribution of isotopologues, giving the bifurcation plots shown in Fig. 2. The model predicts the unlabelled isotopologue of each intermediate to decrease as the proportion of 2 + amu acetyl-CoA entering the cycle increases. Meanwhile, the fully labelled isotopologue increases with rising α. Each of the other isotopologues are predicted to reach their own peak as α increases, decreasing again to 0 as the proportion of [1,2-^13^C_2_]acetyl-CoA entering the Krebs cycle continues to rise.

### Validation of the model

**Fig. 3:**
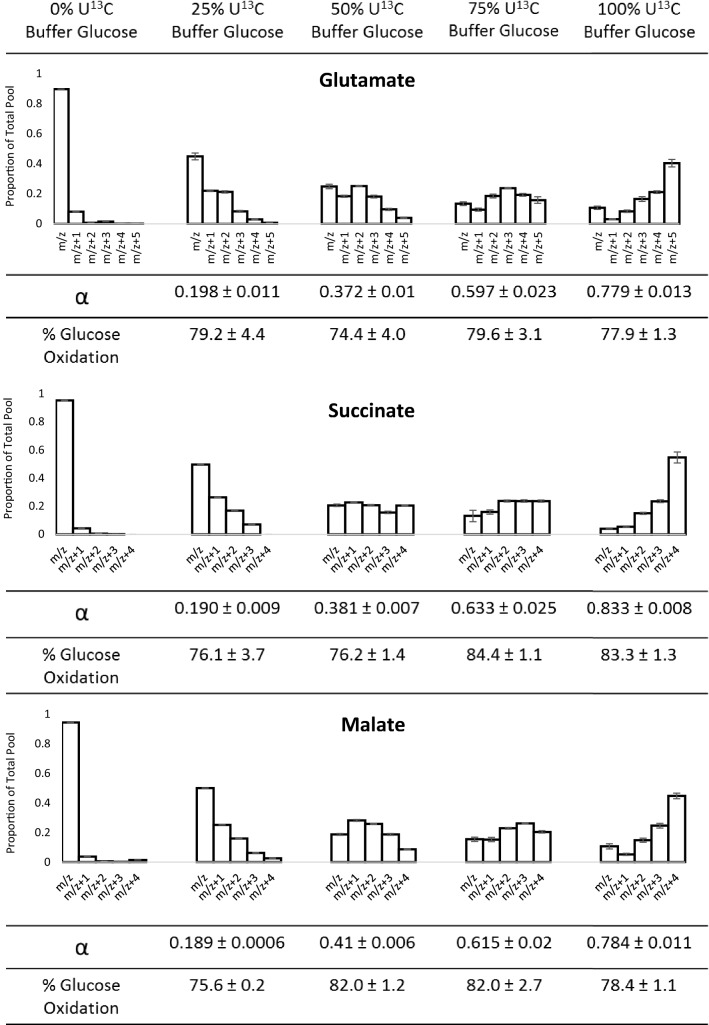
Isotope distribution plots showing the labelling distribution of glutamate, succinate and malate when the percentage of glucose in the perfusion buffer which was U-^13^C labelled was 0%, 25%, 50%, 75% or 100% (each n = 3). Values displayed as mean ± SEM. Note that owing to the small value of the SEM relative to the mean, the SEM is highlighted in grey on some bars

When the proportion of labelled glucose provided in the perfusion buffer was varied, and the resulting steady-state isotopologue distributions determined by LC–MS, α was fitted separately for each experimental replicate, using each of malate, glutamate and succinate independently. Citrate was not considered here because of the higher labelling concentrations used, and the higher concentration of 6 + amu citrate which would result. The value of α assigned to these by the model was largely consistent (Fig. 3), and the discrepancy of fit values all lower than 0.065 (Supplementary Materials). Across three different intermediates, and hearts supplied with four different proportions of U-^13^C glucose, the average calculated contribution of glucose was 79.1% ± 0.9% SEM. 2 way ANOVA indicated the percentage of Krebs cycle oxidation accounted for by glucose oxidation was not affected by the percentage of buffer glucose which was U^13^C labelled, by which intermediate was assessed, or by any interaction between factors (all p > 0.05). Thus, it seems the model is an accurate tool for analysing how [1,2-^13^C_2_]acetyl-CoA is incorporated into the Krebs cycle and is effective across the whole range of labelling inputs irrespective of intermediate measured. The remaining ~ 20% of acetyl-CoA entering the TCA cycle which cannot be accounted for by buffer glucose oxidation most likely stems from catabolism of endogenous unlabelled triacylglycerol and glycogen stores.

### Determination of the relative oxidation of substrates in an isolated heart preparation

**Fig. 4:**
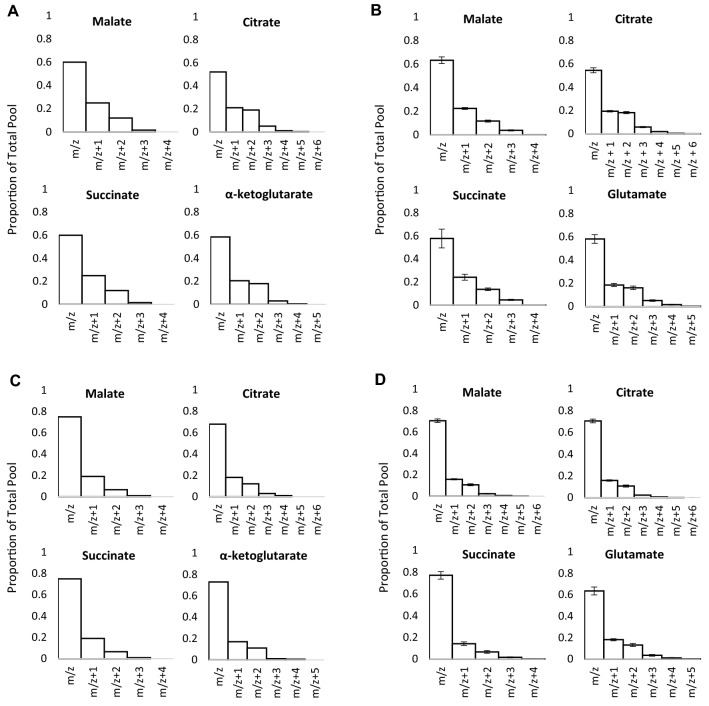
**a** Isotopologue distributions predicted by the model at α = 0.145 for malate, citrate, α-ketoglutarate and succinate. **b** Isotopologue distributions for malate, citrate, glutamate and succinate measured in heart tissue perfused for 32 min with 25% U^13^C labelled glucose by LC–MS (n = 7, displayed as mean ± SEM). **c** Isotopologue distributions predicted by the model at α = 0.089 for malate, citrate, α-ketoglutarate and succinate. **d** Isotopologue distributions for malate, citrate, glutamate and succinate measured in heart tissue perfused for 32 min with 25% U^13^C labelled triglycerides by LC–MS (n = 7, displayed as mean ± SEM). All hearts were perfused with a total of 11 mM glucose and 0.4 mM equivalent triglyceride

When 0.4 mM equivalent Intralipid was added to the perfusion buffer and 25% of the glucose was U-^13^C labelled, the isotopologue distributions shown in Fig. 4b were produced, as measured by LC–MS. Given the agreement of α values fitted from each individual metabolite in Fig. 3, citrate, glutamate, succinate and malate were used simultaneously to fit α. It was appropriate to use citrate because of the low levels of labelling used, and therefore the low level of 6 + amu citrate expected. The observed isotopologue distributions were calculated by the model to most closely fit those predicted when α = 0.145 (± 0.009) (Fig. 4a), a fit which gave the discrepancy score of 0.0139 (± 0.0005). Since only ¼ of the buffer glucose was labelled, relative contribution of glucose to Krebs cycle oxidation is four times 14.5%, at 58.0 ± 3.6%.

The accuracy of the model was then corroborated by determining α when the labelled acetyl-CoA originated from the triacylglycerol in the perfusion buffer. Labelling 25% of the perfusion buffer triacylglycerol resulted in smaller proportions of labelled isotopologues than with the labelled glucose when measured by LC–MS (Fig. 4d). The model determined α to be 0.089 ± 0.002, a 35.6 ± 0.8% contribution of perfusion buffer Intralipid oxidation to total Krebs cycle oxidation. The predicted isotopologue distribution for α = 0.089 again closely matched the observed experimental data, with a discrepancy score of 0.0105 (± 0.0002).

This helps to confirm the validity of the model, as together triacylglycerol oxidation (35.6%) and glucose oxidation (58.0%) account for almost 100% of the acetyl-CoA entering the Krebs cycle in this preparation. The remaining 6–7% of oxidised acetyl-CoA likely corresponds to oxidation of endogenous lipid and glycogen stores. This is a lower figure than the 20% endogenous substrate oxidation observed when no Intralipid was supplied exogenously (Fig. 3), as the heart will preferentially oxidise exogenously supplied fatty acids over endogenous lipid stores (Saddik et al. 1993; Saddik and Lopaschuk 1991). Likewise, fatty acid oxidation is known to inhibit glucose oxidation via the Randle cycle (Hue et al. 2009; Randle et al. 1963), and therefore it is not surprising to see that provision of triacylglycerol results in lower glucose oxidation than when glucose alone was present in the perfusion buffer (Fig. 4b vs. Fig. 3; 58.0% vs. 79.1%). That glucose oxidation exceeds fatty acid oxidation in this Langendorff preparation may reflect the proportions of glucose and Intralipid available to the heart. The 11 mM KH buffer glucose concentration is more than twice that in the blood of a normal rat under fasted conditions (Bell et al. 2011).

### Strengths and limitations of the model

A strength of this model is that it considers entire isotopologue distributions and the fate of individual labelled carbons. Analysis cannot be simplified to the assumption that one specific labelled isotopologue alone signifies greater oxidation of the labelled substrate. Indeed, it is possible to see from LC–MS measurement of isotopologue distribution that as the proportion of the glucose available to the heart which is U-^13^C labelled increases, the proportion of some labelled Krebs cycle intermediates (such as 2 + amu isotopomers) actually decreases. The model illustrates that all levels of 2 + amu intermediates can correspond to more than one value of α (Fig. 2). The unlabelled and fully labelled isotopologues are exceptions, as each concentration corresponds to only one α value. Use of these two isotopologues alone to assess α would be possible, however resolution would be lost because their levels change less rapidly at mid-levels of α. At low α there is also no fully-labelled isotopologue, so if considering these two isotopologues alone it would be possible to falsely conclude that there was no labelling. It is therefore imperative to consider the entire isotopologue distribution rather than specific individual isotopologues.

Consideration of the entire isotopologue distribution also allows us to avoid any impact of natural labelling abundance. As can be seen from Fig. 3, natural abundance of ^13^C labelling in the Krebs cycle results in a very low proportion of 1 + amu labelling and essentially no labelling of 2 + amu or higher. As ^13^C labelling from U-^13^C labelled metabolites increases by an unknown factor, this low level of abundance would decrease by the same unknown factor due to reduced influx of “natural” carbon, making it difficult to correct for. However, since our model gives equal weighting to all isotopologues in the distribution, this minimises the impact of the negligible 1 + amu labelling originating from natural ^13^C abundance.

The model also accounts for migration of labelled carbon within intermediates. This is significant because not doing so may lead to assumptions that carbons in positions not corresponding to the acetyl-CoA which was incorporated in that turn of the cycle were labelled via other pathways. However, our model demonstrates that this labelling can be accounted for by the natural intra-molecular migration of ^13^C originating from acetyl-CoA as the cycle progresses.

The proposed metabolic network model, described in Supplementary Table 1–3, does not include reactions related to pyruvate carboxylation, and hence is not designed to assess flux via this pathway. Negligible anaplerosis is therefore an underlying assumption of this network model. In other organs or metabolic conditions where greater levels of pyruvate carboxylase flux would be expected, there would be a greater level of 3 + amu and heavier isotopologues. A larger discrepancy between our model’s prediction and the shape of distribution in the experimentally-measured dataset would be therefore observed. In the heart, however, even at levels of pyruvate carboxylase flux such as 5–10%, we would expect no significant deviance between the observed isotopologue distributions and those predicted by our model. For other conditions, where anaplerosis is expected to be significant, a more complex model should be considered.

As well as glucose, lactate may act as a source of pyruvate for oxidation in the Krebs cycle (Lloyd et al. 2003). Oxidation of circulating lactate may also be measured using this model. In the experiments described in this paper, no lactate was included in the initial buffer composition, so any present has been generated from pyruvate, and will exhibit an identical isotopologue distribution to that of pyruvate at steady state. Other experiments with an exogenous supply of labelled lactate may behave differently, in which case this model could be employed to determine exogenous lactate oxidation.

An assumption this model makes is that at steady state metabolite fluxes are constant and therefore isotopologue distributions are unlikely to differ between subcellular compartments. Were it to transpire that this is not the case, there would be no impact on the validity of the model, but in order to test mitochondrial metabolites they should be extracted from isolated mitochondria preparations rather than whole tissue.

Back-flux, whereby the reverse reaction of Krebs cycle enzymes occurs, is accounted for in the model because the stoichiometry is the same regardless of reaction direction or rate. For example, if one isotopologue of fumarate precedes 50:50 proportions of a malate isotopologue, the reverse reaction will convert both malate isotopologues back to the same isotopologue of fumarate, so it does not influence isotopologue distribution.

One limitation of the model in its current format is that it assumes all acetyl-CoA is either 0 + or 2 + amu. No 1 + amu acetyl-CoA was used in this work, so this was not needed here, but the model could easily be adjusted to account for this if required.

## Concluding remarks

This mathematical model has been developed for the analysis of ^13^C labelling patterns of Krebs cycle intermediates and has been demonstrated to accurately interpret isotopologue distributions which result from the whole range of different labelling inputs from U^13^C labelled substrates.

Potential applications of the model might include assessment of metabolic substrate preference under different physiological or pathological conditions. One such paradigm would be the failing heart, which is incapable of supplying itself with sufficient blood and oxygen for sustenance of contractile function (Dargie 2005). This is thought to lower overall substrate oxidation, but increase the ratio of glucose:fatty acids oxidised (Neubauer 2007). This model could be used to assess progression of failure via the percentage contribution of glucose oxidation to total substrate oxidation by the Krebs cycle.

## Electronic supplementary material

Below is the link to the electronic supplementary material.

Supplementary material 1 (DOCX 18 kb)

## Data Availability

The datasets generated during and/or analysed during the current study are available in the University of Cambridge repository, 10.17863/CAM.39870. The MATLAB model generated during this study is available in the University of Cambridge repository, 10.17863/CAM.39868.
