# Supplementary material for: A model for determining cardiac mitochondrial substrate utilisation using stable 13C-labelled metabolites
Source: Metabolomics. 2019 Nov 26;15(12):154. doi: 10.1007/s11306-019-1618-y (PMC6892366; doi:10.1007/s11306-019-1618-y)
Supplement: Supplementary file 1 — Supplementary material 1 (DOCX 18 kb) [file 11306_2019_1618_MOESM1_ESM.docx]

**Supplementary Material for:**

**A Model for Determining Cardiac Mitochondrial Substrate Utilisation using Stable ^13^C-labelled Metabolites**

Ross T. Lindsay^1,2^, Demetris Demetriou^3^, Dominic Manetta-Jones^2^, James A. West^1^, Andrew J. Murray*^2^ and Julian L. Griffin*^1^

1 Department of Biochemistry and the Cambridge Systems Biology Centre, University of Cambridge, UK

2 Department of Physiology, Development and Neuroscience, University of Cambridge, UK

3 Department of Engineering, University of Cambridge, UK

* These authors made an equal contribution to the manuscript

**Supplementary Table 1: Atom Transitions in Glycolysis**

| **Molecule Transition** | **Carbon Transition** |
| --- | --- |
| Glucose <-> Pyruvate | ABCDEF <-> ABC + DEF |
| Pyruvate <-> Acetyl CoA | ABC <-> AB |

**Supplementary Table 2: Atom Transitions in β-Oxidation**

| **Molecule Transition** | **Carbon Transition** |
| --- | --- |
| Fatty Acid of Chain Length 2n -> n Acetyl-CoA | (AB)_n_ -> n(AB) |

**Supplementary Table 3: Atom Transitions in the Krebs Cycle**

| **Molecule Transition** | **Carbon Transition** |
| --- | --- |
| Acetyl-CoA + Oxaloacetate -> Citrate | AB + CDEF -> FEDBAC |
| Citrate <-> Isocitrate | FEDBAC <-> FEDBAC |
| Isocitrate -> α-ketoglutarate | FEDBAC -> FEDBA |
| α-ketoglutarate -> Succinyl-CoA | FEDBA -> EDBA |
| Succinyl-CoA <-> Succinate | EDBA <-> EDBA |
| Succinate <-> Fumarate | EDBA <-> EDBA |
| Fumarate <-> Malate | EDBA <-> 50% ABDE + 50% EDBA |
| Malate <-> Oxaloacetate | 50% ABDE + 50% EDBA <-> 50% ABDE + 50% EDBA |

**Supplementary Table 4: Composition of Intralipid**

| ***Constituent*** | **% of Intralipid Represented** |
| --- | --- |
| ***Egg Yolk Phopholipids*** | **1.2%** |
| ***Glycerin*** | **2.25%** |
| ***Soybean Oil***  ***Lipid composition of which is as follows:*** | **20%** |
| *Linoleic Acid* | 44 – 62% |
| *Oleic Acid* | 19 – 30% |
| *Palmitic Acid* | 7 – 14% |
| *Linolenic Acid* | 4 – 11% |
| *Stearic Acid* | 1.4 - 5.5% |

NB. Intralipid is sold as a 20% emulsion**Supplementary Table 5: MSI Parameters**

| **Metabolite** | **m/z** | **Retention time (min)** |
| --- | --- | --- |
| Citrate | 191.0108-191.0278 | 1.75 |
| Citrate +1 amu | 192.0162-192.0300 | 1.75 |
| Citrate +2 amu | 193.0200-193.0330 | 1.75 |
| Citrate +3 amu | 194.0200-194.0400 | 1.75 |
| Citrate +4 amu | 195.0250-195.0450 | 1.75 |
| Citrate +5 amu | 196.0277-196.0461 | 1.75 |
| Citrate +6 amu | 197.0270-197.0380 | 1.75 |
| Glutamate | 148.0301-148.0610 | 0.76 |
| Glutamate +1 amu | 149.0218-149.0370 | 0.76 |
| Glutamate +2 amu | 150.0340-150.0622 | 0.76 |
| Glutamate +3 amu | 151.0242-151.0580 | 0.76 |
| Glutamate +4 amu | 152.0252-152.0352 | 0.76 |
| Glutamate +5 amu | 153.0240-153.0344 | 0.76 |
| Succinate | 117.0157-117.0300 | 2.15 |
| Succinate +1 amu | 118.0191-118.0270 | 2.15 |
| Succinate +2 amu | 119.0224-119.0293 | 2.15 |
| Succinate +3 amu | 120.0256-120.0335 | 2.15 |
| Succinate +4 amu | 121.0303-121.0358 | 2.15 |
| Malate | 133.0096-133.0198 | 1.00 |
| Malate +1 amu | 134.0108-134.0238 | 1.00 |
| Malate +2 amu | 135.0177-135.0250 | 1.00 |
| Malate +3 amu | 136.0211-136.0279 | 1.00 |
| Malate +4 amu | 137.0244-137.0316 | 1.00 |

**Supplementary Table 6: Discrepancy Scores for Fitted α Values from Fig.3**

|  | **25%** | **50%** | **75%** | **100%** |
| --- | --- | --- | --- | --- |
| **Succinate** | 0.0171 ± 0.0006 | 0.0646 ± 0.0031 | 0.0456 ± 0.0113 | 0.0194 ± 0.0012 |
| **Malate** | 0.0192 ± 0.0009 | 0.0166 ± 0.0020 | 0.0432 ± 0.0055 | 0.0396 ± 0.0053 |
| **Glutamate** | 0.0087 ± 0.0002 | 0.0241 ± 0.0007 | 0.0311 ± 0.0032 | 0.0340 ± 0.0043 |
